# Clinical Relevance of *ATRX/DAXX* Gene Mutations and ALT in Functioning Pancreatic Neuroendocrine Tumors

**DOI:** 10.1007/s12022-025-09848-1

**Published:** 2025-02-15

**Authors:** Brenna R. van ´t Veld, Wenzel M. Hackeng, Claudio Luchini, Lodewijk A. A. Brosens, Koen M. A. Dreijerink

**Affiliations:** 1 Department of Pathology, University Medical Center Utrecht, Utrecht, The Netherlands; 2 Department of Diagnostics and Public Health, Section of Pathology and ARC-NET Applied Research on Cancer Center, University of Verona, Verona, Italy; 3 Department of Endocrinology and Metabolism, Amsterdam University Medical Centers, Amsterdam, The Netherlands

**Keywords:** Pancreatic neuroendocrine tumors, Insulinoma, ATRX, DAXX, ALT, Prognostic biomarker

## Abstract

Functioning pancreatic neuroendocrine tumors (PanNETs) represent a subset of PanNETs that cause symptoms due to hormonal activity. Insulinoma is the most common functioning PanNET type. Mutations in the alpha thalassemia/mental retardation X-linked (*ATRX*) and death domain-associated protein (*DAXX*) genes result in genomic instability. *ATRX/DAXX* mutations and associated alternative lengthening of telomeres (ALT) are common in non-functioning PanNETs and associated with aggressive tumor behavior. Recent reports have shown that *ATRX/DAXX* mutations and ALT are also present in functioning PanNETs. In this review, we summarize the literature addressing *ATRX/DAXX* mutations and ALT in functioning PanNETs and discuss the clinical relevance with regard to distinguishing aggressive and indolent functioning tumors. *ATRX/DAXX* gene mutations and/or ALT have been reported in insulinoma, glucagonoma, gastrinoma, VIPoma and calcitoninoma. In insulinoma, the presence of *ATRX/DAXX* mutations and ALT are associated with aggressive behavior and could therefore be used as prognostic biomarkers. Although *ATRX/DAXX* mutation and ALT assessment may currently not be the standard of care in routine diagnostic pathology practice, the use of DAXX/ATRX immunohistochemistry at least can be encouraged not only for non-functioning but also for functioning PanNETs.

## Introduction

Pancreatic neuroendocrine tumors (PanNETs) are rare neuroendocrine neoplasms, occurring in fewer than 1 per 100.000 persons annually. Yet, PanNETs represent approximately 5–10% of all pancreatic tumors and are therefore the second most common solid pancreatic tumor [1, 2]. PanNETs arise from cells in the islets of Langerhans and can be classified into either functioning or non-functioning tumors depending on symptomatic hormonal activity. Non-functioning PanNETs (NF-PanNETs) represent approximately 90% of all PanNETs, are often asymptomatic and frequently diagnosed incidentally [3]. Functioning PanNETs on the other hand, usually present with a clinical syndrome resulting from the autonomous production of hormones. The symptoms vary depending on the type of hormone produced, for example, insulin, glucagon, gastrin, vasoactive intestinal peptide (VIP), adrenocorticotropic hormone (ACTH), parathyroid hormone-related peptide (PTHrP), calcitonin, growth hormone-releasing hormone (GHRH), or somatostatin. The most common type of functioning PanNET is the insulin-secreting PanNET, also called insulinoma [3]. A diagnosis of insulinoma can be suspected by recognizing “Whipple’s triad”: low plasma levels of glucose, symptoms of hypoglycemia, and prompt disappearance of symptoms after the correction of hypoglycemia and diagnosed endocrinologically by detection of inappropriate insulin secretion in a fasting test [4].

The mainstay of treatment of PanNETs is surgical resection, in particular in case of large or symptomatic tumors. According to the European Neuroendocrine Tumor Society (ENETS) and North-American Neuroendocrine Tumor Society (NANETS) guidelines, follow-up with imaging after surgical resection of NF-PanNETs and the majority of functioning PanNET types is recommended until 10–20 years after surgery [5, 6]. For insulinomas, which generally have a more favorable prognosis than other functioning PanNETs, there is no consensus among the guidelines with regard to the need for clinical or radiological follow-up after surgery: the ENETS guideline states that patients with an insulinoma lower than grade 3 (i.e., mitotic rate < 20% mitoses per 2mm2or Ki-67 proliferation index < 20%) without signs of malignancy only need a single follow-up after 3–6 months [6, 7]. The NANETS guideline does not include a recommendation for follow-up [8]. Although insulinomas typically display oncologically indolent behavior, an aggressive course including lymph nodal and distant metastases can occur and is difficult to predict: even small, low-grade insulinomas may metastasize [9, 10]. Furthermore, metastases can develop many years after surgical resection of the primary tumor [11–14]. The 5-year survival of patients with an indolent insulinoma has been reported to be 94–100%; for patients with an aggressive insulinoma, this is significantly lower [4, 7]. Therefore, there is a clinical need for additional prognostic biomarkers that can distinguish aggressive insulinomas with metastatic potential from their indolent counterparts, as patients at high risk of recurrence may benefit from intensified follow-up.

Exome sequencing studies in sporadic NF-PanNETs have identified frequent somatic mutations in genes encoding epigenetic regulators, particularly the alpha thalassemia/mental retardation X-linked (*ATRX*) and death domain-associated protein (*DAXX*) genes [15]. Inactivating *ATRX* or *DAXX* mutations leads to disrupted telomere chromatin stability, triggering alternative lengthening of telomeres (ALT) as well as resulting in chromosomal instability (CIN) and replicative stress [16, 17].

Whereas several studies have emphasized the prognostic relevance of *ATRX*, *DAXX* gene mutations, and ALT in NF-PanNETs, their value as biomarkers of behavior of functioning PanNETs is less well-studied. Therefore, in this review, we summarize the literature addressing *ATRX*/*DAXX* mutations and ALT in functioning sporadic PanNETs with an emphasis on insulinoma. We discuss mechanistic aspects of ATRX/DAXX and the consequences of *ATRX*/*DAXX* mutations, their occurrence in functioning PanNETs, as well as potential future implementations of *ATRX/DAXX* mutations and ALT as prognostic biomarkers for use in clinical decision-making in functioning PanNET patient care.

## ATRX, DAXX, and ALT in PanNET Tumorigenesis

**Fig. 1:**
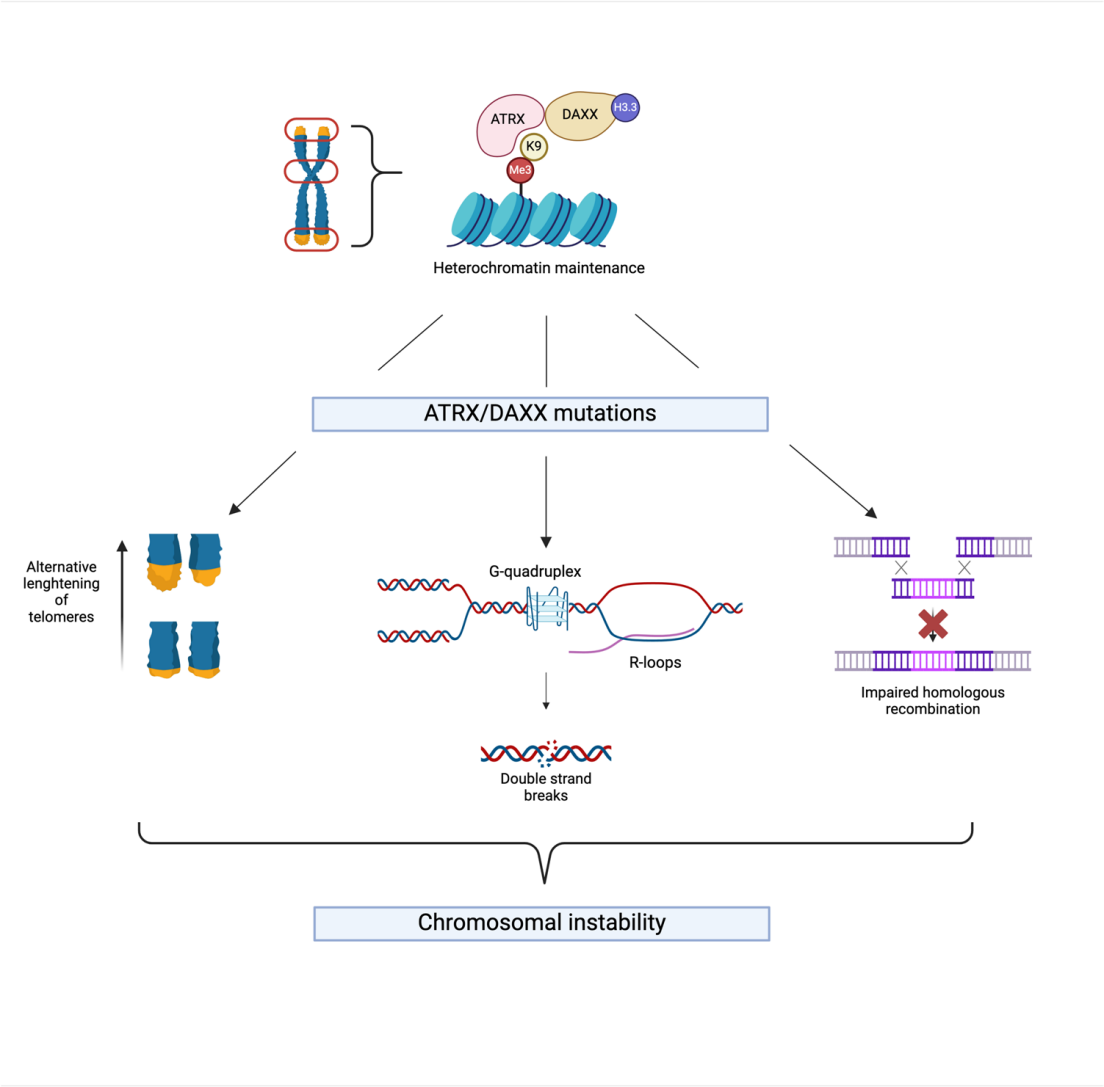
Consequences of *ATRX* and/or *DAXX* mutations. ATRX and DAXX form a histone chaperone complex responsible for the deposition of histone variant histone H3.3 in H3K9me3 enriched repetitive regions of heterochromatin, such as telomeric and pericentromeric regions (red circles, upper section). Mutations in either *ATRX* or *DAXX* have several key consequences leading to or associated with chromosomal instability and tumorigenesis: alternative lengthening of telomeres (ALT, left), double strand DNA breaks due to defective resolution of G-quadruplex (G4) DNA complexes and R-loops (middle), and impaired homologous recombination (right). Created with BioRender.com

In 2011, an exome sequencing study by Jiao et al*.* revealed frequent somatic mutations in the *ATRX* and *DAXX* genes in a series of sporadic NF-PanNETs [15]. This observation was later confirmed using whole-genome sequencing by Scarpa et al*.* [18]. *ATRX* and *DAXX* mutations are mutually exclusive. Interestingly, genetic inactivation of *ATRX* or *DAXX* is associated with CIN, metastatic potential, and significantly shorter survival in sporadic primary PanNETs [19]. These findings suggest that both *ATRX* and *DAXX* play a crucial role in the pathogenesis of PanNETs. Although the exact tumorigenic mechanisms in PanNETs are still unknown, several hypotheses have arisen from research on ATRX and DAXX in essential cellular processes (Fig. 1).

Together, the *ATRX* (chromosome Xq21.1) and *DAXX* (chromosome 6p21) gene products form a histone chaperone complex, responsible for the deposition of histone variant histone H3.3 in repetitive regions of heterochromatin, such as pericentromeric and telomeric regions. In this complex, *ATRX* can bind to histone H3 lysine 9 trimethylation (H3K9me3) enriched heterochromatin [20–22]. It then targets *DAXX* bound to H3.3 to form the interaction required for histone deposition and regulate the maintenance of heterochromatin in these regions.

In 2011, Heaphy et al*.* discovered that inactivating mutations in either *ATRX* or *DAXX* are strongly associated with ALT [17]. ALT is a form of telomere maintenance based on a homology-directed DNA repair mechanism, independent of telomerase activity. It is present in around 5–10% of all cancer types, including epithelial tumors and sarcomas [23]. Of note, it is present with a higher prevalence, approximately 30%, in NF-PanNETs [24–26]. Importantly, *ATRX* and *DAXX* mutations do not appear to initiate tumor development but rather occur at a later stage where they are associated with progression to metastatic disease and poor survival rates [27]. As such, in retrospective studies, ALT and loss of *ATRX* and *DAXX* have been shown to be strong prognostic biomarkers of recurrence or metachronous metastatic disease in sporadic NF-PanNETs [27–29].

Telomere elongation through the ALT pathway is proposed to be triggered by spontaneous double-stranded DNA (dsDNA) breaks, chronic replication stress, or simple telomere erosion [23, 30, 31]. Aberrant telomeric chromatin structure and alterations in the DNA damage repair dynamics that arise as a result of mutations in chromatin remodeling protein-coding genes such as *ATRX* and *DAXX* and DNA damage repair proteins can lead to telomere replication stress [30, 31]. Interestingly, *ATRX* and *DAXX* have also been hypothesized to increase genome stability by facilitating the resolution of G4 DNA structures and thereby avoiding replication stress [32]. Recently, Pinto et al*.* reported *DAXX* to promote genome stability by preventing the accumulation of R-loops during transcription and subsequently preventing DNA double-strand breaks at centromeres in an *ATRX*-independent fashion [33]. Additionally, the complex appears to play an important role in the DNA repair synthesis during homologous recombination (HR), as either *ATRX*- and *DAXX*-deficient cells are unable to repair exogenously induced double-strand breaks (DSBs) through HR [34]. Thus, deficiencies in either protein can lead to an increased number of chromosomal alterations, which in turn can contribute to tumorigenesis.

## ATRX, DAXX and ALT in Insulinomas

Insulinomas are the most prevalent type of functioning PanNETs, accounting for 95% of cases. However, with an incidence of 1–4 per million per year, their prevalence is low [35, 36]. According to the WHO classification from 2022, nonmetastatic insulinomas are referred to as “indolent” and metastatic insulinomas as “aggressive” [37]. The predominant recurring genetic variant in up to 30% of sporadic insulinomas is an activating T372R missense mutation in the Yin Yang 1 (*YY1*) gene, which is absent in NF-PanNET [38]. Jonkers et al*.* reported in a series of 44 indolent and 18 aggressive insulinomas that CIN, defined as arbitrary DNA copy number variations, outperformed tumor size and tumor grade in predicting metastatic potential [39]. More recently, copy number amplifications have been reported in a series of indolent insulinomas [40]. In 2020, Hackeng et al. proposed that CIN in insulinoma might be associated with underlying *ATRX* or *DAXX* mutations, as was shown previously for NF-PanNETs [19, 41]: in a cohort of 35 insulinomas, of which 30 indolent and five aggressive, ALT was observed in four out of five aggressive insulinomas while it was absent in all indolent cases. Of three aggressive insulinomas assessed for ATRX/DAXX protein expression, DAXX loss was found in one case (also ALT positive). None of the indolent insulinomas showed ALT, or ATRX or DAXX loss. Of interest, the aggressive insulinomas displayed aristaless-related homeobox (ARX) expression, which is usually absent in beta-cells and present in alpha cells, suggesting either dedifferentiation or an alpha cell-of-origin mechanism in these tumors. During progression initially non-functioning PanNETs or subclinical glucagonomas expressing ARX could gradually acquire clinically relevant insulin-production [41].

In 2024, Hong et al*.* reported no *ATRX/DAXX* mutations in a large cohort of indolent insulinomas [42]. Several more cases of aggressive insulinomas have been described in the literature. For example, a study by Di Domenico et al*.* included two aggressive insulinomas, of which one was *ATRX*/*DAXX* mutated while the other was *ATRX*/*DAXX* wildtype [43]. Also, separate cohorts from Chan et al*.* and Sadanandam et al*.* both included an aggressive insulinoma case harboring an *ATRX* mutation [44, 45]. In the literature, two additional aggressive insulinomas have been assessed for ALT, of which one tested positive and one negative [18, 43]. As summarized by Hackeng et al*.* in 2023, ATRX/DAXX protein loss and ALT are extremely rare in indolent insulinomas [10]: ATRX/DAXX protein loss or mutation was observed in 3/212 (1%) tested indolent cases, compared to 5/10 (50%) tested aggressive cases. In addition, the ALT phenotype was present in 3/41 (7%) and 5/7 (71%) of tested indolent and aggressive cases, respectively. More recent studies by Zhang et al*.* and Hong et al. reported the absence of *ATRX*/*DAXX* mutations in four and two aggressive insulinomas respectively [42, 46]. The only insulinoma in the cohort of Moser et al*.* that metastasized 4 years after surgical resection did not harbor *ATRX*/*DAXX* mutations either [47]. Recently, Backman et al*.* performed a multi-omics analysis in a series of aggressive PanNETs including one insulinoma case. Interestingly, *ATRX* mutations were found in both the primary tumor and the metastases; however, these were different pathogenic variants [48]. Also, a low-grade insulinoma liver metastasis recently reported harbored an *ATRX* gene mutation [49]. Taken together, these data show that *ATRX* and/or *DAXX* mutations and ALT are mostly absent in indolent insulinomas, while they occur frequently in aggressive insulinomas.

## ATRX, DAXX, and ALT in Other Functioning PanNETs

The remaining 5% of functioning PanNET cases include several types of tumors, classified based on the secreted hormone and consequent clinical syndrome [50]. These include gastrinomas, glucagonomas, VIPomas, ACTHomas, PTHrPomas, calcitoninomas, GHRHomas, and somatostatinomas. As these functioning PanNETs usually have higher malignant potential than insulinomas, the frequency of follow-up according to the ENETS guidelines is every 3–6–12 months, similar to NF-PanNETs [6].

Di Domenico et al*.* found loss of ARTX/DAXX expression in one out of four gastrinomas, in a patient who developed a relapse [43]. Hong et al*.* did not find *ATRX*/*DAXX* mutations in 9 gastrinomas assessed [42]. Five aggressive gastrinomas reported in other studies were wild type regarding *ATRX*/*DAXX* [18, 43, 44, 51]*.* Of interest, PanNET organoid cells were grown from a grade 3 liver metastasis of a gastrinoma harboring a *DAXX* mutation [52]. Recently, a PanNET grade 3 case initially presenting with hypoglycemia and a *DAXX* mutation and later with NEC-like transformation and hypergastrinemia has been reported [53].

Hong et al*.* found *ATRX*/*DAXX* mutations in two glucagonomas, one with lymph node metastasis [42]. In the series of Di Domenico et al*.* aberrant ATRX/DAXX expression was found in one of two glucagonomas that relapsed [43]. Mattiolo et al*.* investigated *ATRX*, *DAXX* mutations, and ALT in six patients with glucagonomas [54]: All patients had lymphatic and vascular invasion upon diagnosis, four out of six had nodal metastases and one already presented with liver metastases. All tumors were single large masses (mean size 8.2 cm): this finding, along with the fact that the symptoms were of recent onset in all patients, may indicate that glucagon secretion was a late feature of such lesions, not present from the beginning. In total, four out of six patients had mutations in either *ATRX* or *DAXX* with subsequent protein loss in the primary tumor (*ATRX* loss in one case, *DAXX* loss in three cases). Four out of six patients developed metachronous metastases, in a wide interval of 24–228 months after initial surgery. Along this line, a case of pancreatic glucagonoma was recently described by Tamura et al*.*, who identified biallelic inactivating mutations in the *DAXX* gene [55]. Additional studies described four aggressive glucagonomas, three of which harbored *ATRX*/*DAXX* mutations [18, 43, 48].

Sadanandam et al*.* included one gastrinoma and also one ACTHoma in their cohort, both negative for *ATRX*/*DAXX* mutations [45]. Pancreatic ACTHomas seem to harbor totally different molecular alterations, being enriched of fusion genes including *EWSR1* rearrangements [56].

Di Domenico et al*.* found aberrant ATRX/DAXX staining in one out of two VIPomas, and the tumor in which the mutation was observed relapsed. Hong et al*.* found no *ATRX/DAXX* mutations in 5 VIPomas [42, 43]. Among three additional reported VIPomas that were tested regarding ARTX/DAXX status, one was found to carry a *DAXX* mutation [18, 43, 44]. Interestingly, Backman et al*.* described one case of aggressive calcitoninoma, where the primary tumor harbored an *ATRX* mutation that was not present in the metastases. All four metastases of this patient carried the same *DAXX* mutation [48]. Pancreas polypeptide (PP) production by pancreatic PPomas is generally not considered to result in a hypersecretion syndrome. To date, there are no published reports addressing *ATRX/DAXX* mutations and ALT in PPomas.

## Conclusions and Future Perspectives

In recent years, evidence has emerged for the role of *ATRX*/*DAXX* mutations and ALT in functioning PanNETs, especially in insulinoma, but also in glucagonoma, gastrinoma, VIPoma, and calcitoninoma. Whereas most insulinomas are considered “indolent” meaning that they usually do not recur or metastasize after resection, around 10–15% of insulinomas may display aggressive behavior and metastasize either locally or to distant organs. In insulinomas, the presence of *ATRX* and *DAXX* mutations, CIN, and ALT is strongly associated with aggressive behavior and is likely prognostic. However, several cases of aggressive insulinomas with no mutations in *ATRX* and *DAXX* and subsequent ALT have also been reported, making it difficult to fully exclude metastatic potential in the absence of these genomic alterations. This underscores the need for further studies to elucidate other pathways contributing to tumor progression such as CIN patterns. Moreover, the prognostic significance of *ATRX*/*DAXX* mutations in rare functioning PanNETs, such as glucagonomas, gastrinomas, and VIPomas, warrants deeper investigation, given the higher malignancy rates in these tumors and potential shared pathogenic mechanisms.

To conclude, incorporating *ATRX*/*DAXX* mutation and ALT status into the diagnostic workflow for insulinomas could significantly improve the identification of patients at higher risk of metastasis. These patients may need more intensive follow-up, similar to NF-PanNETs and other functioning PanNETs. Ideally, insulinomas, both indolent and aggressive, should be more extensively studied to further delineate the relationship between *ATRX/DAXX* mutational status, ALT, CIN, and metastatic spread. Additionally, multi-omics approaches could uncover alternative mechanisms of metastasis in *ATRX*/*DAXX*-wildtype tumors, providing a more comprehensive understanding of their progression. As future prospective studies in patients with this rare tumor type may be challenging, implementation of using *ATRX*/*DAXX* mutations and ALT as prospective biomarkers in real-world prospective registry studies may be a feasible option with direct implications for clinical practice.
